# An update of label-free protein target identification methods for natural active products

**DOI:** 10.7150/thno.68804

**Published:** 2022-01-24

**Authors:** Zhao Cui, Caifeng Li, Peng Chen, Hongjun Yang

**Affiliations:** 1 Institute of Chinese Materia Medica, China Academy of Chinese Medical Sciences, Beijing 100700, China.; 2 Beijing Key Laboratory of Traditional Chinese Medicine Basic Research on Prevention and Treatment for Major Diseases, Experimental Research Center, China Academy of Chinese Medical Sciences, Beijing 100700, China.; 3 Robot Intelligent Laboratory of Traditional Chinese Medicine, Experimental Research Center, China Academy of Chinese Medical Sciences & MEGAROBO, Beijing, China.

**Keywords:** protein target identification, protein stability, label-free methods, natural active products, drug discovery

## Abstract

Natural active products (NAPs) are derived from chemical substances found in nature that have biological activity and medicinal potential. Screening and revealing the protein targets of NAPs is an indispensable link in the pharmacological and toxicological understanding of NAPs. Proteins are the main factors executing cell functions, and cells rely on the function of proteins to complete various activities in the life cycle. The important mechanism of action of drugs is to regulate cell biological activities by interacting with proteins and other macromolecules. At present, the classic way to screen protein targets is based on the molecular label tracing method, which has a long cycle and changes the molecular structure and pharmacological effects of NAPs. Due to the shortcomings of molecular labelling methods, in recent years, scientists have tried to develop a variety of label-free protein target identification methods for NAPs and have made a certain amount of progress. This article reviews the current protein target identification methods for NAPs with the aim of providing a reference for research on NAP protein targets.

## Introduction

More than 200 years ago, people extracted morphine, a natural active product with clear pharmacological activity, from the opium poppy plant for the first time 1. Since then, the era of obtaining drugs from plants began. After the second World War, due to the discovery of penicillin, the scope of drug research expanded to the field of microorganisms 2, 3. By 1990, approximately 80 % of drugs were either natural active products (NAPs) or analogues inspired by them 4. Antibiotics (e.g., penicillin, tetracycline, and erythromycin), antiparasitic drugs (e.g., avermectin), antimalarials (e.g., quinine and artemisinin), lipid regulators (e.g., lovastatin and its analogues), organ transplantation immunosuppressants (e.g., cyclosporine and rapamycin) and anticancer drugs (e.g., paclitaxel and doxorubicin) have revolutionized medicine. Analysis has shown that more than one-third of the 1881 new drugs approved from 1981 to 2019 were directly or indirectly derived from NAPs; among synthetic drugs, approximately half of the drugs borrowed the skeleton structure or pharmacophore of NAPs 5. Using NAPs with clear pharmacodynamics as molecular probes, discovering their targets of action and clarifying their molecular mechanism are conducive to discovering active lead structures with clear targets and clear mechanisms of action, providing a scientific basis for the development of innovative drugs. At the same time, the discovery of the protein targets of NAPs helps identify potential drug targets for future drug design and activity screening. Therefore, research on natural active product (NAP) protein targets is conducive to promoting the development of innovative drugs.

Targets are biomolecules related to diseases that interact with NAPs and have special sites that match active small molecules. Target recognition is the process of identifying the direct molecular target of a compound. Active small molecules induce various physiological reactions in cells, organs, tissues or body states by interacting with biomolecules to form complexes that play an important role in complex regulation and exert certain therapeutic effects on pathological conditions 6. Studies have found that drug targets can typically be classified in six main categories: enzymes, cell surface receptors, nuclear hormone receptors, ion channels, transporters, and DNA. Proteins are the main regulators of life activities, and in most cases, the macromolecular targets of NAPs are proteins 7. Therefore, the identification of protein targets of NAPs is an important issue in the process of chemical biology research and the development of new drugs.

A regulatory mechanism that is composed of small molecule-target-phenotype can regulate various activities in the body. According to the logical relationship between molecules, targets and phenotypes, the strategies for identifying NAP protein targets are divided into two categories 8. One is the direct target identification strategy based on NAPs; the other is the indirect target identification strategy based on phenotype. Phenotype-based indirect target identification strategies indirectly infer the direct target and action mode of the compound based on known information, such as phenotypic changes at the multigroup level and the corresponding cellular signal pathways. The direct target identification strategies are methods for screening protein targets that is focused on NAPs. Different research mechanisms are usually divided into chemical labelling methods based on chemical proteomics and label-free methods based on protein stability 9. The chemical labelling methods based on chemical proteomics are the most widely used and are generally divided into affinity-based target identification and activity-based protein profiling 10. Affinity-based target identification relies on the transient binding of a protein to active small molecules combined with solid carriers. These methods are roughly divided into conventional affinity-based pull-down approaches 11, photoaffinity-based approaches 12, and photoaffinity labelling-fluorescence difference in two-dimensional gel electrophoresis (FITGE) approaches 13. These methods usually include two key steps: (1) design and synthesis of chemical probes and (2) target capture and recognition. Because the protein targets captured by small molecules will be washed repeatedly and the buffer solution will be exchanged during the experiment 14, strong molecular cis-binding ability between small molecules and protein targets is needed. Therefore, medium or weak binding of small molecules to proteins is difficult to identify. Derivative modification of NAPs with complex structures may reduce or lose the original activity; in addition, a large number of non-specifically binding proteins will be introduced in the process of affinity purification. Activity-based probes (ABPs), such as activity-based protein profiling (ABPP), have been developed for protein target recognition in chemical proteomics to overcome the shortcomings of affinity-based target strategies. This method uses an activity-based probe to specifically label functional proteins in the proteome to reflect the functional state of proteins 10, 15. Compared with the traditional affinity-based protein profiling (AfBPP) and compound-centric chemical proteomics (CCCP) strategies 16, 17, the greatest advantage of the competitive ABPP strategy is that it does not require tedious probe synthesis 10, 18, and thus it is particularly suitable for complex NAPs with a low natural abundance that are difficult to chemically modify and may avoid the effects of chemical derivatives on the structure and activity of NAPs. However, ABPP also has limitations; for example, the existing active probes are limited to the detection of only a few amino acid residues, such as cysteine and lysine, and are suitable only for NAPs that react with these amino acid residues. In summary, the modification of active molecules or target proteins requires a full understanding of their structure-activity relationships. Although the covalent binding of the probe to the target protein expands the scope of protein target screening to small molecules with low binding affinity, slight modifications may also affect the interaction between the original molecule and the target protein, resulting in target recognition errors and failures.

Although chemical labelling methods based on chemical proteomics have been widely used and developed, they cannot be used for rapid and unbiased protein target identification of small molecules with a tight structure-activity relationship or complex NAPs due to the need for probe synthesis or molecular modification. In recent years, researchers have developed a series of label-free screening methods for protein targets that do not require the structural modification of small molecules and are based on the biophysical properties related to changes in protein stability. The mechanism for these methods is that when a small molecule binds to its protein target, the thermal stability, proteolysis or chemical stability of the protein will change, thereby revealing the protein target of NAPs. The degree of this transformation strongly depends on the characteristic properties of the protein targets. Importantly, compared with chemical proteomics methods, label-free methods based on changes in protein stability efficiently screen protein targets in a mixture, so they are particularly suitable for the rapid identification of protein targets of NAPs.

Many reviews have discussed the development and application of drug protein target identification methods 9, 19-24. Here, we focus on reviewing the latest developments in direct and indirect label-free screening methods for protein targets of NAPs, discussing the advantages and disadvantages of each strategy, and providing a future outlook on their scope of application and development trends.

## Direct label-free strategies for identifying protein targets of NAPs

**Figure 1:**
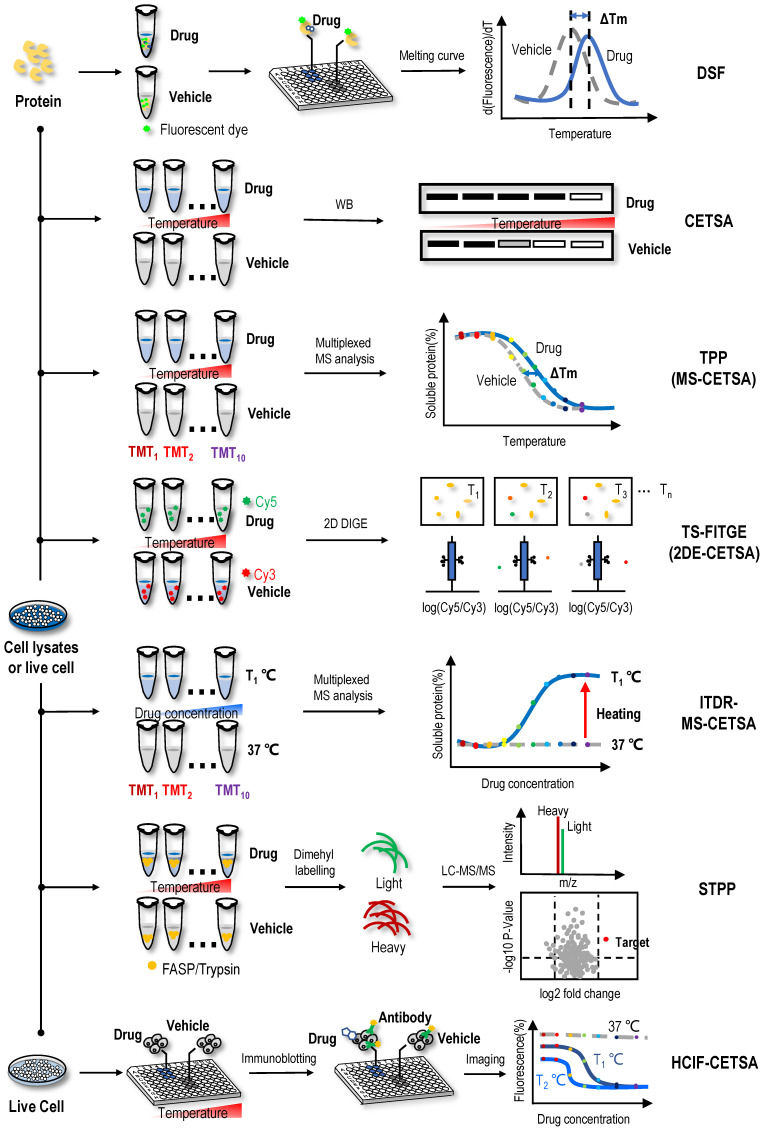
** Label-free target identification methods based on shifts in the thermal stability of protein targets.** When the proteins are heated, their folded structures denature, and the proteins begin to aggregate. Proteins have intrinsic properties of resistance to thermal denaturation, which can be described by the Tm, the temperature when half of the proteins is denatured. Interactions between proteins and small molecules can alter their free energy and thermal stability. Label-free target identification methods based on shifts in the thermal stability of protein that include DSF, CETSA, TPP (or MS-CETSA), TS-FITGE (or 2DE-CETSA), ITDR-MS-CETSA, STPP and HCIF-CETSA.

**Figure 2:**
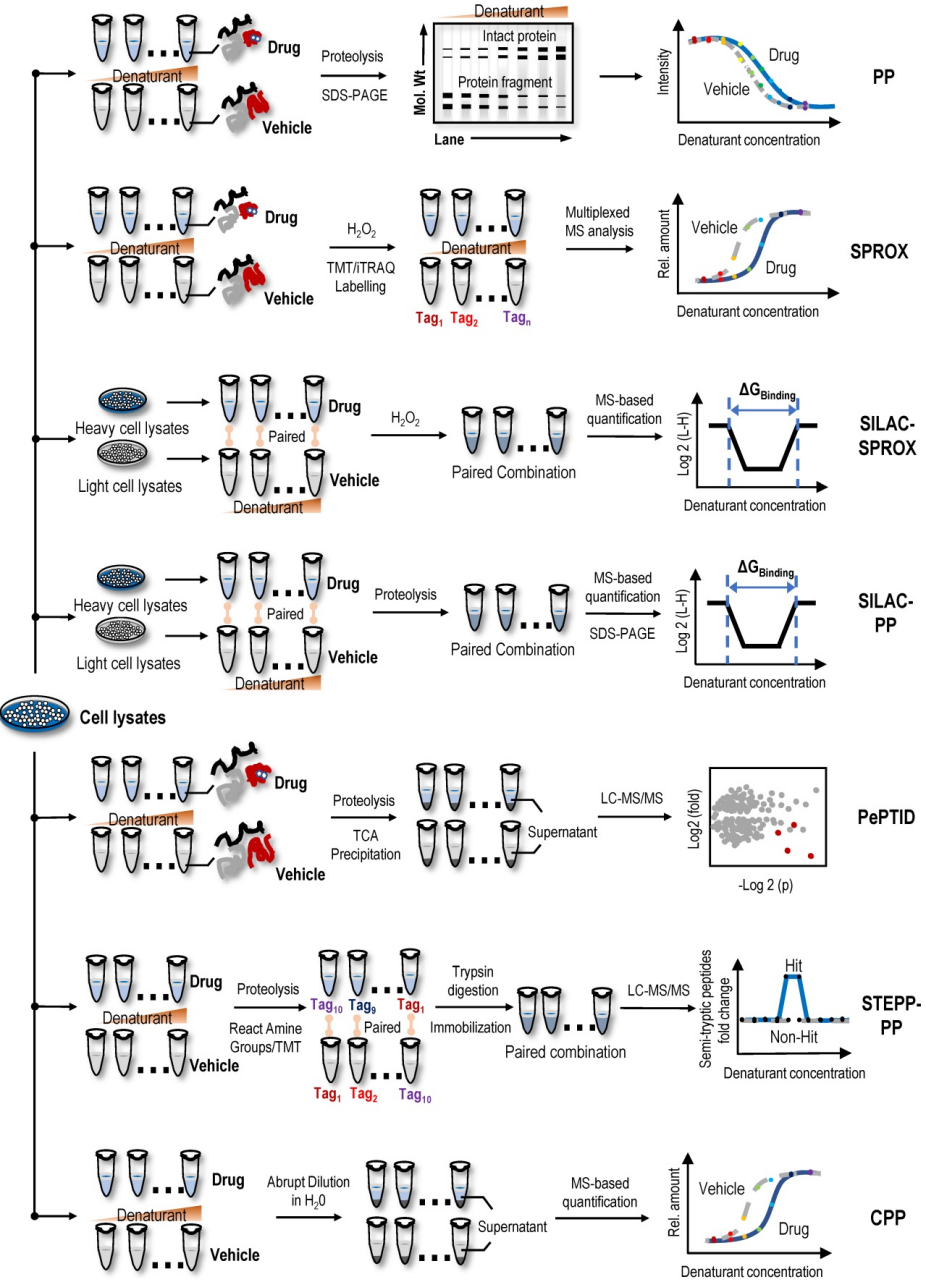
** Label-free target identification methods based on the difference in chemical denaturant-induced stability of protein targets.** Proteins can be denatured by chemicals (i.e., denaturants), such as guanidine salts or urea. Denatured proteins are more susceptible to proteolysis or oxidation than intact proteins. The stability of protein to denaturants can be changed by the combination of small molecules, so as to shift the proteolytic stability and oxidation level of protein. Label-free target identification methods based on the difference in chemical denaturant-induced stability of protein that include PP, SPROX, SILAC-SPROX, SILAC-PP, PePTID, STEPP-PP and CPP.

**Figure 3:**
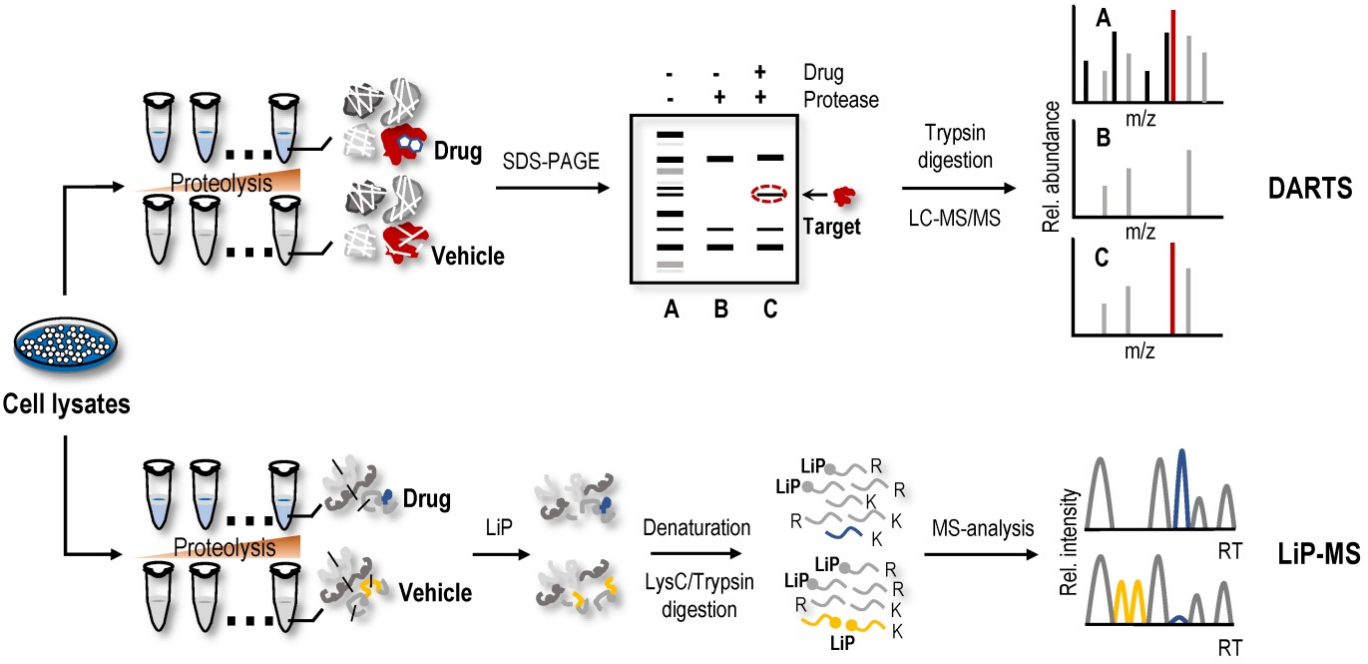
** Label-free target identification methods based on the shift in the limited proteolytic susceptibility of protein targets.** The protein conformation is influenced by a variety of factors, including post-translational modifications, disease state, and ligand binding, which may alter the proteolytic susceptibility. DARTS and LiP-MS are label-free target identification methods based on the shift in the limited proteolytic susceptibility of protein.

**Figure 4:**
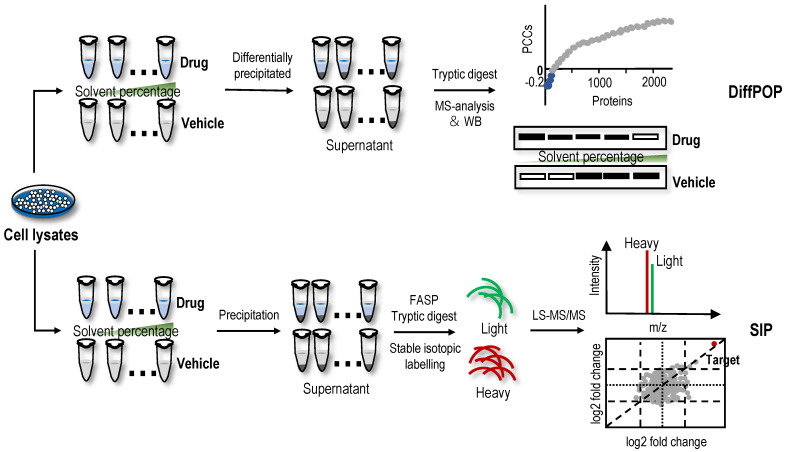
** Label-free target identification methods based on organic solvent-induced shift in the solubility of protein targets.** Organic solvents are commonly used to precipitate proteins and remove contaminants. The resistance of the protein targets to organic solvent-induced denaturation and precipitation is stronger after ligand-binding events. DiffPOP and SIP are label-free target identification methods based organic solvent-induced shift in the solubility of protein.

**Figure 5:**
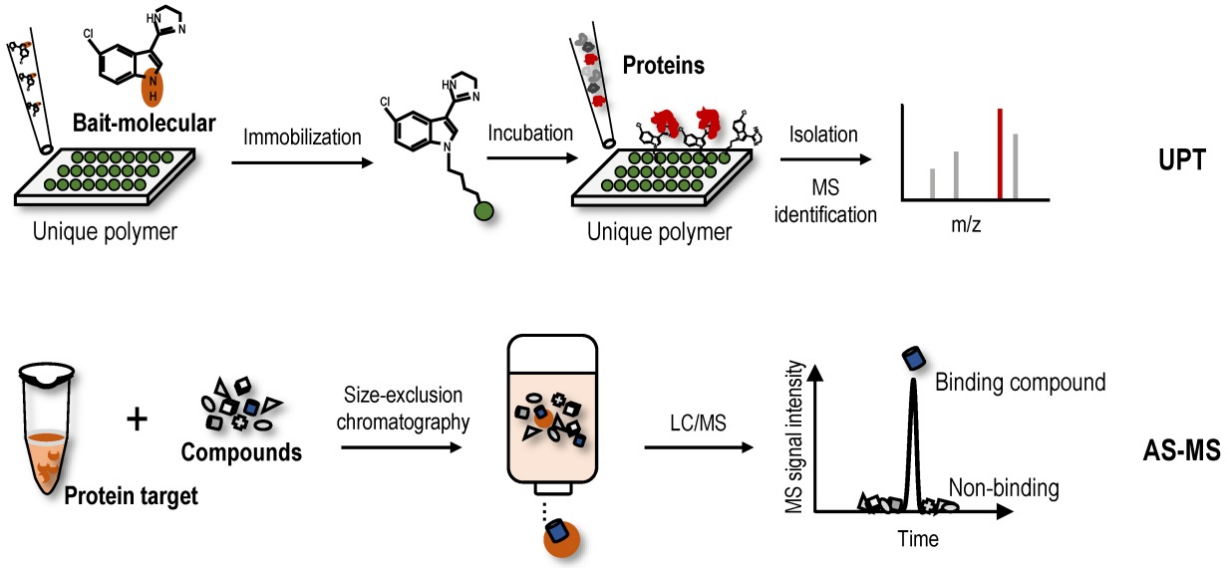
** New label-free screening strategies.** UPT uses the weak molecular interaction of bait-molecule to non-covalently immobilize it on a polymeric surface to achieve target protein fishing. AS-MS is an affinity-based screening technique for the analysis of interactions between protein targets and small molecules.

The binding of small molecules to protein targets might influence the stability of the target proteins, such as shifts in the thermal stability, chemical denaturant-induced stability and proteolysis susceptibility of the proteins. The strategies for directly identifying protein targets of NAPs include methods for screening protein targets of NAPs as a starting point. Therefore, based on the biophysical properties of changes in protein stability, researchers have developed a series of methods for screening protein targets without the structural modification of small-molecule drugs. Examples include cellular context thermal shift assays (CETSA) based on differences in protein thermal stability 25-27; the pulse proteolysis (PP) method and stability of proteins from rates of oxidation (SPROX) method based on differences in protein chemical denaturant-induced stability 28, 29; the drug affinity responsive target stability (DARTS) method based on differences in proteolysis susceptibility 30; and solvent-induced protein precipitation (SIP) based on differences in protein solubility 31. In this section, we will introduce direct label-free methods based on the physical and chemical properties of NAPs and differences in protein stability induced by various factors and review the application of these methods in the identification of NAP protein targets (Figure 1-5).

## Direct screening strategies based on the physical and chemical properties of NAPs

NAPs have various structures that contain conjugated structure systems and often exhibit ultraviolet (UV) absorption; therefore, measuring the intensity of UV absorption is a common method for detecting these NAPs. Based on the unique UV absorption spectrum of an unknown compound, a new natural product, 4-(4-dihydroxymethylphenoxy) benzaldehyde, which is a derivative of p-phenoxy-benzaldehyde in bamboo shoots, was identified 32. In addition, the existence of functional groups, such as large conjugated systems, molecular cyclization and benzene rings, endows some NAPs, such as resveratrol and chlorogenic acid, with fluorescent properties and these molecules autofluoresce upon excitation with a certain wavelength of light 33-35. The autofluorescence of NAPs can be used to study the utilization and absorption of NAPs by cells and the cellular localization of NAPs. Importantly, combining the autofluorescence of NAPs with two-dimensional gel electrophoresis (2D DIGE) and mass spectrometry (MS) technology facilitates the rapid identification of the protein targets binding to NAPs and the subsequent study of the molecular mechanism. For example the uptake profiles of resveratrol and piceid were studied in cancer cells based on autofluorescence properties using fluorescence microscopy and confirmed using liquid chromatography-tandem mass spectrometry (LC-MS/MS) 36. Our research group utilized the autofluorescence of chlorogenic acid to exploit its protein targets and found that chlorogenic acid binds annexin A2, causing a decrease in the expression of anti-apoptotic genes downstream of NF-κB 37. The method detecting the autofluorescence of NAPs overcomes the limitations of traditional small molecule labelling strategies and substantially improves the efficiency and accuracy of target identification. However, these methods are limited to NAPs with UV absorption and fluorescence spectra, and many NAPs do not have obvious UV absorption and fluorescence spectra.

## Direct screening strategies based on the differences in the thermal stability of protein targets

### Differential scanning fluorometry (DSF)

Natural proteins should be folded in a thermodynamically favourable manner with the lowest energy to function normally in cells. When the environmental temperature is greater than the activation energy of the protein, denaturation occurs under the control of the entropy factor 38, 39. The thermal stabilization technology of protein targets is based on the shift of the denaturation curve with temperature after the protein target and ligand bind, which can be expressed as the difference in the degradation temperature and difference in trends of the melting curve of protein targets (Figure 1). DSF is a convenient method to evaluate the thermal stability of proteins under a range of conditions 40. Based on this method, a medium- to high-throughput platform has been established to discover small-molecule stabilizers of protein targets for drug discovery 41. In DSF experiments, the hydrophobic part of the protein rather than the protein itself has a unique affinity for a fluorescent dye. These hydrophobic parts are exposed and bound to the fluorescent dye as the protein unfolds when the temperature increases; thus, researchers only need to mix the protein of interest with the detection dye and candidate stabilizer, heat the sample in a controlled manner, and record the fluorescent signal as a function of temperature to obtain the melting curve for the protein under a range of conditions 42, 43. By comparing the melting temperature (Tm) of the protein unfolding transition in the melting curve, the binding of ligand and protein can be analysed.

Using DSF methods, inhibitor VIII, a commercially available PH domain-dependent allosteric serine/threonine-protein kinase 1/2 (AKT1/2) inhibitor, resulted in a dose-dependent increase in the Tm of AKT1, suggesting that AKT1 bound to inhibitor VIII and that the binding stabilized the protein 44. Rigosertib is a non-ATP-competitive inhibitor of PLK1, and one study subjected recombinant proteins to the DSF method in the presence of rigosertib to determine the site to which rigosertib binds and found that the RAF-RAS-binding domain bound to rigosertib, as indicated by the change in Tm 45. In another study, changes in thermodynamic stability of the N-terminal domain of *Arabidopsis* clathrin heavy chain-1 (CHC-1) occurred in the presence of endosidin9, a potential endocytosis inhibitor, in a concentration-dependent manner; and the protein stability of the N-terminal domain in the presence of endosidin9 was similar for *Arabidopsis* CHC-1, clathrin heavy chain-2 (CHC-2) and human CHC-1 46. Altogether, these results indicated that endosidin9 binds to the N-terminal domain of CHC in both *Arabidopsis* and humans 46. By studying the ligand-binding properties of MtCuvA using DSF, MtCuvA was shown to bind to the cell wall precursor components uridine diphosphate (UDP) -glucose and UDP-N-acetyl-glucosamine 47. Another study used the DSF method and found that the binding of the bioactive substance pacFA ceramide to P53 caused the Tm of p53 to increase from 41.6 °C to 43.2 °C 48. However, no standard is available for determining the extent to which the Tm value changes to determine the occurrence of the ligand-binding event, so this method has a certain degree of subjectivity. Recently, high-throughput DSF screening was performed to screen NAPs that potentially bind and modulate the stability of an oncogenic microRNA from the molecular targets program (MTP) pure compound library; found that the natural product butylcycloheptyl prodiginine specifically bound to precursor microRNA-21 (miR-21) to inhibit its processing into mature oncogenic miR-21 and selectively arrest the growth of colon cancer cells 49. Although DSF is technically feasible and promising for identifying ligand-binding events, a great deal of destabilization occurs in DSF experiments, such as ligands covalently modifying the protein, saturation of receptor-ligand binding, changes in ionic strength leading to a depletion of ions that stabilize the protein, dosage of fluorescent dye, or detergent-like denaturation. In addition, the applicability of DSF has been limited to purified proteins *in vitro*. Importantly, the binding of the ligand might not protect the protein from thermal denaturation. In this case, other methods must be employed to study the interaction.

### CETSA

Based on the principle of ligand-induced thermodynamic stabilization of protein targets, a CETSA was developed to evaluate the protein target engagement of drug molecules in cells and tissue samples 27. In this method, intact cells are heated to a range of temperatures in the presence or absence of drug molecules. Then, the cells are lysed, and the soluble fraction is collected. Western blotting (WB) is performed to reveal whether the proteins are denatured in a temperature-dependent manner and whether the melting curves of some proteins binding drug molecules in live cells are shifted (Figure 1). For example, using a CETSA, an obvious shift of ca. 5 ℃ was observed in the Class III PI3K (Vps34) melting curve in the presence of a derivative of the natural product aurone, indicating that the aurone derivative 1a engaged and stabilized Vps34 in cell lysates 50. In the process of screening signal transducer and activator of transcription 3 (STAT3) inhibitors among NAPs using a CETSA and other biochemical methods, 2′-hydroxycinnamaldehyde was found to directly bind to STAT3, inhibiting STAT3 activity 51. Similarly, through the combined use of a CETSA, molecular docking and molecular dynamics simulations, the natural sesquiterpene coumarin ferulin C was shown to bind to the colchicine site of tubulin 52. In addition to the results described above, many studies have used a CETSA alone or in combination to identify protein targets of NAPs. For example, the natural product 10,11-dehydrocurvularin was found to directly interacts with STAT3 using a CETSA 53, the interactions between the natural product geranylnaringenin and SH2 domain-containing protein tyrosine phosphatase-2 (SHP-2) were identified through the combined use of a CETSA and pull-down assays 54, the furanocoumarin isoimperatorin was determined to exert an inhibitory effect on NA-mediated progeny virus release through neuraminidase (NA) inhibition assays and a CETSA 55, and the interactions between nucleolin (NCL) and the active natural product curcumol were validated through the combined use of a CETSA, molecular docking assay and cell-based assays 56.

In addition, a CETSA is often used to confirm the protein targets of solvent extracts of natural plants. For example, the methanol extract of *Melicope accedens* was shown to interact with spleen-associated tyrosine kinase (Syk) using CETSA 57, the methanol extract of the leaves of *Olea europaea* was found to interact with TGF-β-activated kinase 1 (TAK1) using overexpression and CETSA technologies 58, and the interactions between the methanol extract of *C. subulatum Guillaumin* and proto-oncogene tyrosine-protein kinase (Src) and Syk were confirmed using WB and CETSA 59. Compared to DSF, CETSA performs well in versatile sample types, including living cells and tissues. Thus, it is advantageous for compounds that require cellular metabolism for activation and might reveal downstream effects of these compounds when applied to live cells. However, a CETSA is not suitable to detect some proteins containing unfolded binding sites.

### Isothermal dose-response fingerprint-cellular context thermal shift assay (ITDRF-CETSA)

ITDRF-CETSA, a modified version of the CETSA, was proposed to better reflect the dose-dependent interaction of ligands and protein targets 27. In the ITDRF-CETSA, a range of drug concentrations were applied at a constant system temperature to determine whether the protein target binds to the ligands in a dose-dependent manner 60. By performing an ITDRF-CETSA at a constant temperature of 72 °C, an interaction between the antipyretic analgesic acetaminophen and the off-target protein N-ribosyldihydronicotinamide:quinone reductase 2 (NQO2) was detected in the presence of approximately 1 mM extracellular concentrations of the drug, consistent with the *in vitro* substrate assay with a km value of ∼0.4 mM 61. Because NQO2 was predominantly present in the liver and kidney, where it is known to cause toxicity, NQO2 may also have a role in acetaminophen-induced superoxide production *in vivo* 61. Due to the low throughput of ITDRF-CETSAs, a chemical proteomics approach using kinobead was utilized to evaluate 226 clinical kinase inhibitors for their ability to bind the enzyme ferrochelatase (FECH), and 29 of these compounds exhibited low or submicromolar FECH binding 62. Subsequently, K562 cells treated with increasing concentrations of a particular drug were heated to 55 ℃, followed by nondenaturing cell lysis and the confirmation of target engagement in cells 62. The advantage of the ITDRF-CETSA is that it is able to determine the affinity of the drug-protein interaction. Conventional methods, such as isothermal titration calorimetry (ITC) 63, require the use of purified proteins, which are time-consuming, and in some cases, purified protein cannot be obtained. However, an ITDRF-CETSA facilitates the determination of the affinity of a drug with a protein target only using cell lysates where WB readout is available. Overall, both the CETSA and ITDRF-CETSA represent simple and reliable methods for the analysis of protein targets of NAPs. However, they also have some shortcomings, such as the low throughput of target engagement, unsuitability for proteins expressed at low levels, difficulty in detecting some proteins containing unfolded binding sites, and antibody availability. Thus, both the CETSA and ITDRF-CETSA are often used to validate ligand-binding events rather than screen targets.

### Thermal proteome profiling-cellular context thermal shift assay (TPP or MS-CETSA) and isothermal dose-response mass spectrometry-cellular context thermal shift assay (ITDR-MS-CETSA)

With the application of MS technology in protein thermal stability assays, a series of high-throughput protein target identification methods have been proposed, such as TPP (or MS-CETSA) and ITDR-MS-CETSA. By combining the CETSA with multiplexed quantitative MS, TPP was first established for the proteome-wide determination of protein thermal stability in intact cells 64. In the TPP assay (Figure 1), the samples are heated to a range of temperatures in the absence or presence of drug. Then, unlike a CETSA, which directly detects the protein targets using WB, the heat-treated sample is digested into peptides using the standard bottom‐up proteomics sample preparation protocol and labelled with tandem mass tag (TMT) isobaric tags before high‐resolution MS analysis. After qualitative and quantitative analyses using tandem mass spectrometry, the abundance of TMT-conjugated peptides at each temperature is determined according to their reporter ions. The melting curve of each protein is plotted according to the abundance of comprising peptides, and the Tm of the protein is obtained in the absence and presence of drug 64. Then, proteins with a significant Tm shift are selected as target candidates 64, 65. By monitoring the effects of small-molecule ligands on the profiles, researchers delineated more than 50 targets for the kinase inhibitor staurosporine in the TPP assay, they identified the haem biosynthesis enzyme FECH as a target of kinase inhibitors and suggested that its inhibition caused the phototoxicity observed for vemurafenib and alectinib 64. In the subsequent discussion of the protein target profiles obtained using TPP and kinobead assays, experimental data indicated that these two methods measured different protein populations but were potentially complementary 66. The cytotoxic natural product vioprolide A has prominent potency against human acute lymphoblastic leukaemia cells 67. One study used the TPP method and found that nucleolar protein 14 (NOP14), which is essential for ribosome biogenesis, was the target protein of vioprolide A in Jurkat cells 67. One study mapped the proteome-wide protein targets of artone, a natural product from the medicinal plant *Artemisia giraldii*, with a significant anti-neuroinflammatory effect 68. Among the hotspots targeted by artone, a protein target showing the highest isotopic ratio was identified as anti-silencing function 1A histone chaperone (ASF1A). Subsequently, CETSA and siRNA assays confirmed that ASF1A was a direct cellular protein target engaged by artone 68. As an upgraded version of the CETSA method, TPP might provide a general overview of the proteomic state, or proteotype, and might reveal downstream effects when the drug is administered to live cells. However, TPP also has some disadvantages, such as inaccuracy for some proteins with a very low or high Tm and difficulty detecting membrane proteins and some proteins containing unfolded binding sites.

In an ITDR-MS-CETSA (Figure 1), the samples are mixed with a range of drug concentrations at a constant system temperature and then centrifuged to obtain soluble protein. After obtaining the soluble protein, the next MS detection procedure is similar to TPP 69. Proteins with significant differences in isothermal curves at a heating temperature versus at 37 °C (control group) indicate the occurrence of protein target engagement 69, 70. The first implementation of the ITDR-MS-CETSA method was for protein target identification in *Plasmodium falciparum*, the main causative agent of malaria in humans 70. This study first validated the efficacy of this approach for model drugs pyrimethamine, a folic acid antagonist, and E64d, a broad-spectrum cysteine proteinase inhibitor. Then, combining studies in the *P. falciparum* parasite lysate and intact infected red blood cells, they identified *P. falciparum* purine nucleoside phosphorylase (PfPNP) as a common binding target for quinine and mefloquine 70. ITDR-MS-CETSA was also applied in another study of *Plasmodium falciparum* 69*.* Combined with an ITDR-MS-CETSA, an experimental workflow for target screening of antimalarial drugs was designed. The experimental workflow involved treatment of *P. falciparum*-infected erythrocytes with a compound of interest, heat exposure to denature proteins, soluble protein isolation, enzymatic digestion, peptide labelling with TMT, offline fractionation, and LC-MS/MS analysis. This experimental workflow can be used with cellular extracts, live cells *in vitro*, and tissues *in vivo*, thus enabling the drug-protein interaction to occur in a more physiologically relevant environment. Since only one temperature with significant precipitation was analysed, the use of an ITDR-MS-CETSA was simpler than that of a TPP. However, to date, no relevant studies have been reported on the use of an ITDR-MS-CETSA to identify protein targets of NAPs.

### Thermal stability shift-based fluorescence difference in two-dimensional gel electrophoresis (TS-FITGE or 2DE-CETSA) and simplified thermal proteome profiling approach (STPP)

In addition to integration with multiplexed quantitative MS analysis (Figure 1), CETSAs have been combined with 2D DIGE in a strategy named TS-FITGE (or 2DE-CETSA) 71, 72. Briefly, cell lysates are treated with vehicle or the test drug and then heated to a range of temperatures. After centrifugation, proteins in supernatant are labelled with different fluorescent dyes, for example, cyanine-3 (Cy3) for vehicle-treated samples and cyanine-3 (Cy5) for drug-treated groups. Then, the two groups are mixed and separated using 2D DIGE according to their isoelectric point and molecular weight. The ratio of the Cy5 to Cy3 fluorescence signal in each protein spot is quantified by an automated image analysis, and protein spots with outlier ratios (Cy5/Cy3 ratio < 1 or > 1) are considered target candidates and excised for protein identification using MS 71. Because fluorescent dyes label proteins rather than drugs, TS-FITGE is a label-free method. In addition, fluorescent dyes are added after the incubation of proteins and drugs, so it does not interfere with the occurrence of ligand-binding events. In one study, because the drug molecule SB2001, a cytotoxic agent on HeLa cells, lacks chemical modification sites, the combination of TS-FITGE and TPP methods were applied to characterize its mechanism of action 73. Interestingly, the potential protein target leukotriene A4 hydrolase (LTA4H) showed only a marginal Tm shift in the TPP assay, but one isoform of LTA4H displayed a significant thermal shift in the TS-FITGE assay. Therefore, the TS-FITGE method has the potential to identify relevant protein targets when a compound modulates their specific proteoforms 73. In another study, a proteome-wide TS-FITGE was developed to identify proteins exhibiting a shift in thermal stability due to interactions with a small compound. Using TS-FITGE, the anticancer compound NPD10084 was found to induce thermal destabilization of pyruvate kinase M2 (PKM2); NPD10084 disrupted the PKM2 complex and inhibited downstream signalling 72. However, sample fractionation on two-dimensional (2D) polyacrylamide gel is required and only a limited region of the gel (i.e., specific molecular weight range) can be practically excised and analysed; thus, the problem of biases in proteome coverage still exists.

STPP, a simplified version of TPP, was proposed recently (Figure 1). The dimethyl labelling technique is used instead of TMT labelling (in the TPP assay) to quantify proteins, and peptides derived from the same protein are used to determine significantly changed proteins in one LC-MS run 74. Known targets of the model drugs methotrexate and geldanamycin validated the utility of this method 74. Furthermore, some ATP-binding proteins that have not been reported previously were detected in 293T cell lysates, which provided supplementary information for the map of ATP-binding proteins 74. During this experiment, only three temperatures (66 °C, 69 °C and 72 °C) were selected for the quantitative proteome analysis to validate known targets of geldanamycin, exceeding the commonly used temperature range in TPP; thus, it is found that the temperatures for TPP should not be limited to the 37~67 °C range 74. The temperature depends on the actual melting temperature of proteins of interest, which could be revealed by WB if targets are known 74. STPP effectively reduces the reagent cost and MS time, suggesting that it is more suitable for the rapid screening of NAP protein targets and verification of candidate targets screened by other methods, such as affinity chromatography and other energetics-based chemical proteomics methods for NAP protein target identification. However, due to the use of single one-dimensional LC-MS/MS analysis, one disadvantage of this approach is the low proteome coverage; typically, approximately 2000 proteins are quantified at each temperature.

### Immunofluorescence-cellular context thermal shift assay (HCIF-CETSA)

In the latest development of the CETSA method, studies used immunofluorescence staining with antibodies and combined CETSA with high-content microscopy. HCIF-CETSA is a new method that uses high-content, high-throughput single-cell immunofluorescence detection to determine the protein target level after heating adherent cells in a 96-well plate (Figure 1) 75. The HCIF-CETSA method appeared robust and showed a good correlation in target engagement measured using this method and CETSAs for the selective checkpoint kinase 1 (Chk1) inhibitor V158411 75. At the same time, a CETSA protocol in live A431 cells for mitogen-activated protein kinase 14 (MAPK14) was proposed, and the remaining soluble protein was detected *in situ* using high-content imaging in 384-well microtiter plates 76. As a model system, a pilot screen identifying a novel class of small-molecule drugs binding the MAPK14 protein was performed, and these results were then confirmed using a kinase activity assay based on isolated recombinant p38α 76. Antibody-based imaging-CETSAs selectively distinguished between the folded form and aggregated or denatured form of the protein and enabled single-cell quantification of target engagement *in situ* with the possibility of monitoring subcellular localization 77. Because antibodies were used in the detection process, one drawback of using immunofluorescence for target detection is that the number of high-quality, well-validated antibodies available is much lower than that for WB. In addition, due to protein denaturation caused by cell fixation, the antibody may not be able to recognize the protein in its natural state, but the epitope is exposed after the protein is partially denatured during the fixation process. Therefore, choosing antibodies that faithfully report the protein state may be critical for HCIF-CETSAs.

## Direct screening strategies based on the difference in chemical denaturant-induced stability of protein targets

### PP

Proteins denatured by chemical denaturants, such as guanidinium salt or urea, are more susceptible to proteolysis or oxidation than intact proteins. Thus, the increase in protein stability upon ligand binding also reflects the increased resistance to protein denaturation 28. PP utilizes the chemical denaturant-induced shift in protein stability to screen protein targets 28. In the PP method (Figure 2), the samples are treated with a range of concentrations of chemical denaturants (i.e., urea or guanidinium chloride) to form an equilibrium mixture of folded and unfolded proteins. Unfolded proteins are then evaluated by protein cleavage with the treatment of the ''pulse'' of an excessive amount of protease such as thermolysin. Subsequently, the remaining folded proteins are separated by sodium dodecyl sulfate-polyacrylamide gel electrophoresis (SDS-PAGE), and the band intensities of the proteins are quantified. During data processing, the quantification of folded proteins is plotted against the concentrations of denaturant, and proteins with significant midpoint denaturant concentration (Cm) shifts are selected as target candidates. In proof-of-principle experiments, the PP method was used to monitor the change in Cm of maltose binding protein in the presence and absence of maltose, which confirmed the feasibility of this method 28. In summary, PP is a simple diagnostic tool for detecting specific phenotypes related to changes in protein stability, but due to the limitations of antibodies in the detection process, its throughput and sensitivity are low. As the first protein target identification method based on differences in protein stability, although it has some shortcomings, it provides inspiration and reference for subsequent label-free protein target research.

### SPROX

Utilizing specific chemical modification reactions may facilitate an evaluation of the surface exposure or accessibility of certain reactive amino acids of the unfolded protein. Based on these characteristics of surface amino acids, the SPROX method was used to evaluate protein denaturation by measuring the methionine oxidation levels of proteins upon treatment with hydrogen peroxide 78. Briefly, two cell lysate samples are treated with various concentrations of guanidinium chloride, a protein denaturing agent, in the absence or presence of drugs. The methionine residues exposed by denaturation are then oxidized by H_2_O_2_. Subsequently, proteins are digested into peptides, and the oxidized methionine residues of peptide fragments are analysed using MS (Figure 2). The amount of oxidized methionine residues of peptide is plotted against the concentrations of guanidinium chloride, and proteins with a significant Cm shift are selected as target candidates 78. In the first application, the model drug resveratrol, a biologically active ligand with less well-understood protein targets, confirmed the validity of the SPROX method, and in the subsequent experiment, cytosolic aldehyde dehydrogenase (ALDH) and six other potential new proteins were identified as the primary targets of resveratrol using SPROX 78. In subsequent research, the validity of the SPROX method was further confirmed in an experiment designed to identify the well-known yeast protein target models of cyclosporin A, a cyclic nonribosomal peptide composed of eleven amino acids 29. At the same time, a number of studies have also shown the feasibility of the SPROX method by examining the interactions of model drugs with known targets and detected both the on- and off-target effects of protein-drug interactions 29, 79-81.

Following the development of the SPROX method, the combined use of quantitative proteomics technologies, such as isobaric tags for relative and absolute quantitation (iTRAQ), and SPROX achieved the simultaneous analysis of ligand binding to hundreds of proteins in complex biological samples and simultaneously detected the on-target and off-target effects of ligand binding 82. However, this protocol is a discovery platform that requires additional experiments to verify the hit proteins and determine whether the hit proteins result from direct or indirect binding interactions 82. In a study designed to identify the protein targets of the antihistamine clemastine using the global thermal and chemical protein denaturation profiling, clemastine induced a thermodynamic shift in the stability of the *Plasmodium* t-complex 1 (TCP-1) ring complex delta subunit, suggesting an interaction with this protein subunit 83. SPROX in combination with a stable isotope labelling with amino acids in cell culture (SILAC-SPROX) approach was used to compare the equilibrium folding/unfolding properties of proteins in the absence and presence of target ligands and successfully characterized the ATP interactome in yeast 84. In another study using the SPROX and PP techniques, Y-box binding protein 1 (YBX1) was validated as a direct binding target of tamoxifen 85. Despite these successful examples, PP- and SPROX-based target identification methods have limitations, as both are applicable only to cell lysates and not to living cellular systems 81. In addition, SPROX is only applicable to proteins containing methionine residues 86.

### Stable isotope labelling with amino acids in cell culture-pulse proteolysis (SILAC-PP) and pulse proteolysis and precipitation for target identification (PePTID)

Recently, a method termed SILAC-PP coupled the PP method to SILAC quantitation using bottom-up proteomics to improve assay sensitivity 87. In the absence or presence of drugs, labelled heavy or light lysates are mixed after PP treatment. The mixed samples are loaded and separated using SDS-PAGE, and the gel bands showing significantly different intensities are excised and analysed using MS 87. Unlike the PP method, SILAC involves fractionation of samples on SDS-PAGE gels, so it more effectively detects the binding of proteins in whole cell lysates to target ligands. However, only a limited area of the gel is excised and analysed using SILAC-PP; therefore, the problem of biases in proteome coverage still exists. In one study using the SPROX method and SILAC-PP method, over 1,000 proteins in a lysate of MDA-MB-231 cells grown under hypoxic conditions were shown to interact with the natural active product manassantin A 88. Through further analysis, 28 protein hits were identified, and only two of the protein hits were identified using both experimental approaches 88. These results showed that the SPROX and SILAC-PP methods have different biases and the combination of the two methods may play a complementary role in the identification of potential protein targets. At the same time, a new technology, PePTID, related to PP have also been proposed. As a proof-of-concept (Figure 2), PePTID was successfully applied to identify ATP-binding proteins in *Mycobacterium smegmatis* 89. Briefly, the PePTID method uses trichloroacetic acid (TCA) to remove the partially digested and undigested proteins from digested peptides before a simple proteolytic pulse, and the results are analysed and compared using a label-free semiquantitative MS strategy that does not require electrophoresis, substantially improving the throughput.

### Semitryptic peptide enrichment strategy for proteolysis procedures-pulse proteolysis (STEPP-PP) and chemical denaturant and protein precipitation (CPP)

STEPP-PP combines a chemo-selective enrichment technique named semitryptic peptide enrichment strategy for proteolysis procedures (STEPP) with the PP method to advance the analytical depth of domain-level binding information from multidomain proteins 90. This strategy involves reacting the ε-amino groups of the lysine side chains and any N-terminus produced in a limited proteolysis reaction with isobaric mass tags (Figure 2). The samples are then digested with trypsin, and N-hydroxysuccinimide (NHS)-activated agarose resin is used to perform a chemoselective reaction on the N-terminus of the newly exposed trypsin polypeptide to remove the trypsin polypeptide from the solution, leaving only the limited proteolytic reaction to produce a nontrypsin cleavage site semitrypsin peptide for subsequent LC-MS/MS analysis 90. In proof-of-principle experiments, cyclosporin A and geldanamycin were used as model systems to evaluate the performance of STEPP-PP. Interestingly, in the geldanamycin and cyclosporin A binding experiment, the known binding targets were successfully identified as hits, and the protein false negative rate was 0% 90. Compared to the PP method, STEPP increased the number of semitryptic peptides detected in PP experiments by 5~10 times. SETPP-PP also enabled the quantitative determination of ligand binding affinities. For example, using the STEPP-PP method, a Kd of ~7.5 nM geldanamycin-HSP82 binding was obtained, which was consistent with the previously reported value of 9 nM 90, 91. Furthermore, the combination of the STEPP protocol and PP technology generates domain- and amino acid-specific structural information about protein conformational properties 90.

CPP, a cross between the SPROX and TPP techniques, uses centrifugation to precipitate unfolded proteins from solution following the abrupt dilution of the denaturant equilibrated sample (Figure 2); subsequently, the fraction of folded proteins in the supernatant or unfolded proteins in the precipitate is quantified using standard quantitative proteomics as a function of the denaturant concentration 92. Similar to STEPP-PP, the well-known protein target models of cyclosporin A and geldanamycin were used to confirm the validity of the CPP method, and then this method was used to successfully identify protein targets of sinefungin, a broad-based methyltransferase inhibitor 92. Compared to the SPROX technique, the CPP technique improved the proteome coverage by approximately 50% and substantially reduced the false discovery rate. In another study performing a comparative analysis of MS-based proteomic methods for protein target discovery using a one-pot approach, the direct comparison of four methods, SPROX, PP, CPP, and TPP, was performed using the model drug cyclosporin A in yeast cell lysates 93. Benefits to using all four strategies for protein target discovery were identified, including increased proteomics coverage and a false-positive rate close to 0%. Moreover, the one-pot strategy greatly reduced reagent costs and instrument time 93.

## Direct screening strategies based on the differential limited proteolytic susceptibility of protein targets

### DARTS

The protein macromolecules in solution are in dynamic equilibrium among ensembles of conformational states. Protein conformation may be affected by ligand binding, which may alter proteolytic susceptibility 94, 95. Earlier studies have shown this characteristic of proteins, namely, proteins, tends to be more resistant to proteolysis when bound to a ligand 96, 97. Based on the limited proteolysis susceptibility shift of protein targets, label-free drug protein target identification strategies, such as DARTS, were first proposed 30. Briefly, cell lysates are treated with proteolytic enzymes in the absence or presence of drugs (Figure 3). Then, proteins in cell lysates are separated on SDS-PAGE gels, and the protein bands showing enhanced resistance to proteolysis are excised and analysed using MS to identify potential protein targets 30.

The DARTS method is widely used to screen protein targets of NAPs. Using DARTS and ITC methods, daurisoline, a bis-benzylisoquinoline alkaloid isolated from *Rhizoma Menispermi*, was shown to target heat shock protein 90 (HSP90) directly 98. Using the DARTS method, vascular endothelial growth factor receptor 2 (VEGFR2) was identified as a protein target of voacangine, a natural product extracted from *Voacanga africana*, *Trachelospermum jasminoides*, or *Tabernaemontana catharinensis* 99. JaponiconeA, a natural product isolated from *Inula japonica Thunb*, has shown good anti-multiple myeloma potential 100. One study identified possible targets and the mechanism of japoniconeA using RNA-seq and c-Map databases and confirmed direct binding of japoniconeA to NF-κB inhibitor kinase beta (IKKβ) using CETSA and DARTS methods 100. 2'-Hydroxycinnamaldehyde, a natural product isolated from the stem bark of *Cinnamomum cassia,* was found to selectively inhibit STAT3 activity by directly binding to STAT3 using a pull-down assay, DARTS and CETSA experiments 51. Using proteomics analysis and DARTS methods, cysteine-rich angiogenic inducer 61 (Cyr61) was identified as a potential target for rotundifuran, a natural product isolated from *Vitex trifolia L*, in cervical cancer cells 101. In the process of screening NAPs that inhibit STAT3 using DARTS and CETSA methods, geranylnaringenin was found to reduce luciferase activity in a dose-dependent manner and then regulate STAT3 activity by interacting with SHP-2 54. In addition to the aforementioned examples, many studies have used the DARTS method to identify NAP targets 102-106. However, similar to SILAC-PP and TS-FITGE, only a limited area of the gel is excised and analysed using DARTS, so the problem of biases in proteome coverage still exists. With the gradual application of the DARTS method, increasingly improved versions are gradually being proposed to screen the protein targets of NAPs. When identifying protein targets of laurifolioside and dichloroacetate, drug molecules were used to treat living cells instead of cell lysates to advance the DARTS method 107, 108. 2D DIGE was used for the separation and visualization of protein targets to increase the resolution of DARTS 109, 110. Because the method relies on the intuitive recognition of different intensities of protein bands on SDS-PAGE gels, the actual sampled proteome space for DARTS analysis was limited to relatively high abundance proteins. Recently, DARTS samples were directly analysed using MS without gel electrophoresis to increase protein coverage, enabling the identification of protein targets of NAPs such as cryptotashinone and arctigenin 111, 112.

### Limited proteolysis-mass spectrometry (LiP-MS)

A method called LiP-MS combined with LC-MS/MS, utilizing biophysical principle of DARTS, and achieved protein target identification at the proteome level 113. In this method (Figure 3), the proteins undergo two-step digestion 113, 114. The first step is a short-term partial digestion of the proteins with a nonspecific protease, such as proteinase K, at a low enzyme to substrate ratio, called limited proteolysis (LiP). The second step is complete digestion by trypsin. Due to the limited protease accessibility of LiP sites in the ligand-bound protein compared to the ligand-free protein, which would be altered, the peptides with LiP sites are cleaved into two semitryptic peptides in the ligand-free protein, while the same peptides are preserved intact in the ligand-bound protein. Therefore, these peptides with significant changes in abundance may participate in the structural changes in the protein induced by the ligand-binding event 114. Compared to the results of gel-based DARTS, the proteome coverage and assay sensitivity of LiP-MS are significantly increased, and continuous advancements in MS instruments and data acquisition methods still provide room for improvement. Combining LiP with a machine learning‐based scoring framework, the LiP-Quant method was used for target identification in complex proteomes that were substantially enriched for direct drug targets in both human cell lines and yeast, with similar efficiency to other proteome-wide-scale techniques 64, 115, 116, and successfully identified the *B. cinereal* homologue of casein kinase 1 (Bcin06g02870) as the target of the fungicide BAYE‐004 117. In another study, a method that interfaced STEPP with the LiP method (named STEPP-LiP) to isolate the semitryptic peptides generated in MS-based proteome-wide applications of LiP methods not only increased the number of semitryptic peptides detected in the LiP experiments by 5- to 10-fold but also increased the amount of structural information gleaned from limited proteolysis experiments. One report related to LiP-MS in the identification of NAP targets is available. In this case, utilizing DARTS, targeted limited proteolysis coupled to multiple reaction monitoring mass spectrometry (t-LiP-MRM), molecular docking and *in vitro* assays identified crellastatin A, a marine metabolite from the sponge *Crella* sp., as an inhibitor of poly (ADP-ribose) polymerase-1 (PARP-1) 106. Although LiP-MS and DARTS methods are technically feasible and promising for identifying ligand binding regions, their binding affinity and protein target susceptibility to proteolysis are limiting factors 9.

## Direct screening strategies based on the organic solvent-induced difference in the solubility of protein targets

### Differential precipitation of proteins (DiffPOP)

Organic solvent protein precipitation is mainly attributed to two reasons, i.e., a decrease in protein solubility resulting from a reduction in the dielectric properties of the solution and destruction of the hydration membrane of the protein 118-120. Thus, acetone, ethanol, methanol and acetonitrile are commonly used to precipitate proteins and remove contaminants. The ligand-protein complex has a lower energy state, so more energy is required for the unfolded protein than the free protein. Therefore, in principle, the resistance of the protein targets to organic solvent-induced denaturation and precipitation is stronger after ligand-binding events. Based on this principle, DiffPOP was proposed and successfully identified serine hydroxymethyltransferase 2 (SHMT2) as a direct target of the histone demethylase inhibitor JIB-04 121. Subsequently, a SHMT2 knockdown experiment revealed that the depletion of SHMT2 recapitulated the effects of JIB-04 on HIV-1 Tat protein levels, validating the results of the DiffPOP method 121. In the DiffPOP method, the stepwise addition of 90% methanol and 1% acetic acid is used to differentially precipitate proteins into ten fractions. Proteins in the ten fractioned pellets are then washed with cold acetone, solubilized and digested in a standard tryptic digest. The digested samples from the different precipitates are then assessed using MS proteomics analysis (Figure 4). Using a Pearson's correlation coefficient (PCC)-based analysis, proteins with PCCs less than 0 were selected as target candidates. However, in this proof-of-principle experiment, the histone demethylases targeted by JIB-04 were not detected using the DiffPOP approach because they remained soluble in the highest levels of methanol tested in these experiments 121. Therefore, this study reminds us that when the solubility of the target protein in the organic solvent is high, the difference in the solubility of the protein may not be detected using this method.

### SIP

Recently, a new method with a similar principle to DiffPOP named SIP was proposed 19. Briefly, after the cell lysate is incubated with or without drugs, the proteins are denatured with a final percentage of organic solvent ranging from 9% to 19% by adding a mixture of acetone/ethanol/acetic acid at a ratio of 50: 50: 0.1. The soluble proteins are then quantified using stable isotopic dimethyl labelling-based proteomics. The proteins with consistently quantified changes over 2-fold in two replicates are considered the target candidates (Figure 4). In proof-of-principle experiments, the feasibility of the SIP method was validated by detecting the known protein targets of methotrexate, SNS-032 and staurosporine, which are pankinase inhibitors, in cell lysates 19. Using the SIP method, three known proteins of the HSP90 family and several potential off-target proteins, including NADH dehydrogenase subunits NADH: ubiquinone oxidoreductase core subunit V1 (NDUFV1) and NADH: ubiquinone oxidoreductase subunit AB1 (NDUFAB1), were successfully identified for the first time, and NDUFV1, which may be an off-target protein responsible for inducing the side effects of staurosporine, was verified using SIP-WB. By comparing SIP and TPP methods for screening protein targets of staurosporine, experimental data showed that although TPP had wider protein coverage (7767 proteins identified using TPP vs. 1854 proteins identified using SIP), protein targets identified using the different precipitation approaches were complementary 19. In addition, the SIP method can also determine the drug-protein affinity in whole cell lysates using a dose-response assay, and the affinity of the geldanamycin-HSP90AB1 complex evaluated using the SIP approach was consistent with previously reported Kd values 19. In summary, the SIP method represents a good platform for the identification of protein targets of drugs to better understand their side effects and mechanisms of action. However, the standard definition for screening target proteins using the SIP method is arbitrary. Loosening filtering criteria will improve the sensitivity of target protein identification but also has the risk of increasing false-positive identifications. Similarly, when the target proteins have high solubility in high-concentration organic solvents, those protein targets are difficult to detect using the SIP method. In addition, to date, an application of DiffPOP and SIP methods for the identification of NAP protein targets has not been reported.

## Other new label-free screening strategies-Unique polymer technology (UPT) and affinity selection-mass spectrometry (AS-MS)

UPT is significantly different in principle compared to the label-free methods based on the differential stability of protein targets, because this method involves noncovalently immobilizing a bait molecule on a unique polymeric surface through weak molecular interactions with bait protein targets. Although weak, the force provided by the polymeric surface is sufficient to immobilize the bait molecule. Based on this technology, a UPT-based protein target identification method was proposed 122. After incubation with biological lysates, the protein targets were enriched in the prepared bait molecule-specific affinity matrix. Proteins were then eluted from the polymer using a higher concentration of test molecule as elution buffer, and eluted proteins were identified using MS (Figure 5). In the UPT study, bisindolylmalemide-III, a well-established GSK3-β inhibitor, was immobilized on the polymeric surface, and the specific capture of GSK3-β was confirmed using WB; β-Actin-like protein 2 (ACTBL2), neuron-specific enolase (ENO2) and macrophage migration inhibitory factor (MIF) were then identified as putative targets of 5-arylidenethiazolidinone, a novel potent anticancer compound 122. In another study using the UPT method, histone deacetylase 2 (HDAC2) and prohibitin 2 (PHB2) were identified as targets of spiro [pyrrolidine-3,3'-oxindole], a potential anti-breast cancer compound 123. However, due to the high false-positive rates of UPT, its application is limited.

AS-MS is also a label-free method that combines size-exclusion chromatography with liquid chromatography-mass spectrometry (LC/MS) 124. In the AS-MS method, numerous compounds are treated with one protein, and the reaction mixture is then subjected to size-exclusion chromatography (Figure 5). Small compounds that do not interact are retained in the column, while larger molecules that interact with the protein target pass through with bound compounds. Bound compounds are then dissociated and identified using LC/MS 124. With the development of AS-MS, several studies have developed novel label-free target identification technologies for small molecules combining AS-MS technology with an *in vitro* expressed human protein library 125, 126, referred to as open reading frame (ORF) expression/AS-MS. Using these methods, novel protein target interactions with the tumour-vascular disrupting agent vadimezan/ASA404 and diuretic mefruside were reported 125. In addition, as an example of an anticancer agent target, glucose transporter 1 (GLUT8) was identified as a target of SW157765, a member of the ''prodrug'' compounds in which high expression of cytochrome P450 family 4 subfamily F member 11 (CYP4F11) is predictive of and required for the cellular response 126. In the latest research, by combining size exclusion chromatography with high-resolution native MS, researchers achieved quickly identify NAPs of human drug targets using crude natural product extracts without fractionation, which significantly increased the efficiency of target-based NAP drug discovery workflows 127. AS-MS technology is a simple, accurate, and universal label-free binding assay technology that can be applied in high-throughput drug discovery and protein target identification 128. Furthermore, AS-MS technology can be used to classify compound binding sites and determine the dissociation rate constant of compounds 129, 130.

## Indirect screening strategies for protein targets

**Figure 6:**
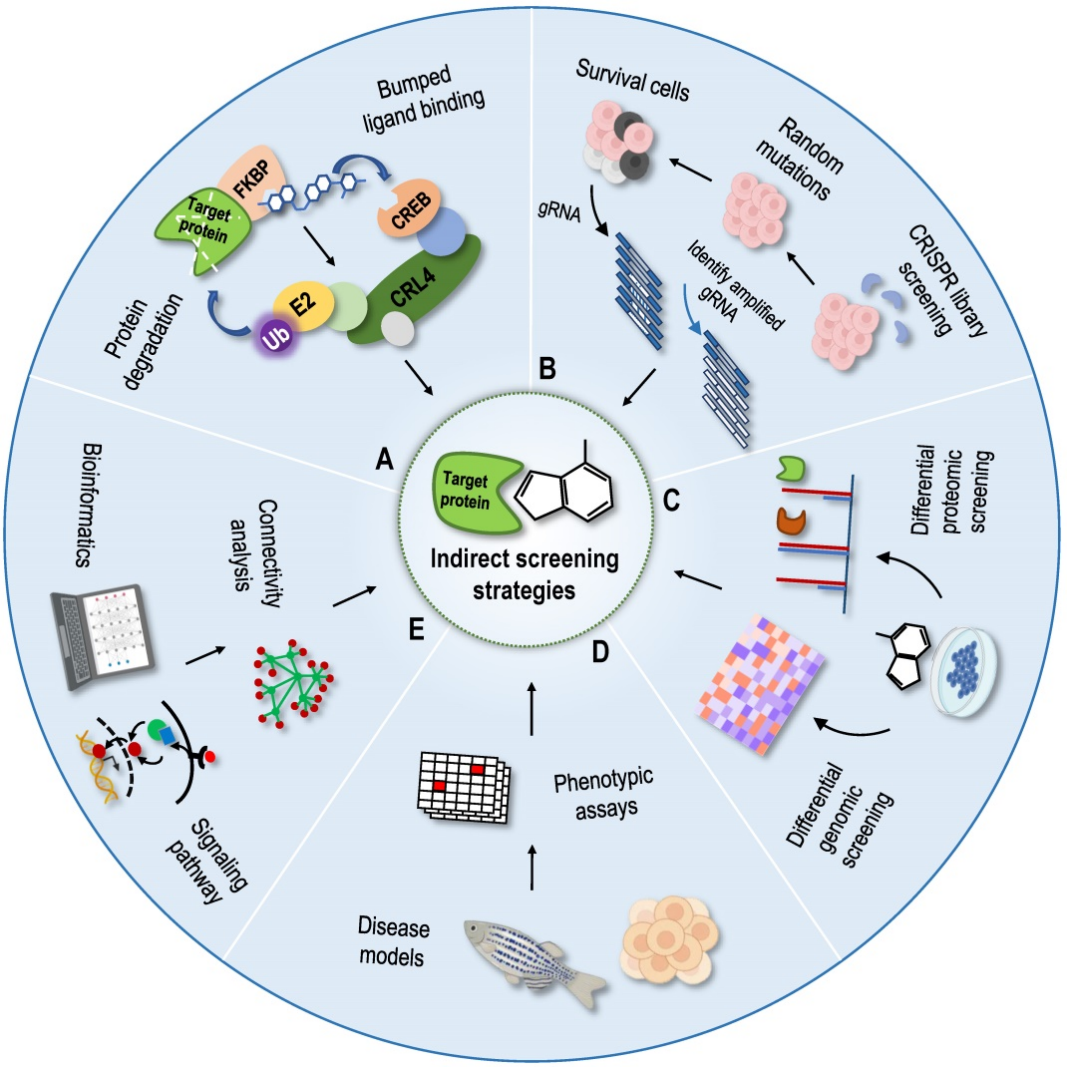
** Indirect screening strategies for protein targets. (A)** Protein degradation methods as dTAG method; **(B)** Genomic library screening methods as the CRISPR genomic library screening; **(C)** Differential genomic screening methods and differential proteomic screening methods; **(D)** Phenotypic-based screening methods; **(E)** Bioinformatics prediction methods.

Phenotype-based indirect target identification strategies are methods that indirectly infer protein targets and action modes of compounds from known information, such as phenotypic changes at the multigroup level and changes in the corresponding cellular signal pathways. The related phenotypic screening methods mainly include protein degradation methods 131, genomic library screening methods 132, differential genomic screening methods 133, differential proteomic screening methods 134, and cell morphological comparison methods 135-137. With the development of structural biology and computational chemistry, as well as the establishment of various connectivity map databases, bioinformatics analysis methods have been gradually applied to identify NAP protein targets 138-140. According to the principle of graph matching, the relationships among drugs, genes and diseases have been established using a large database of characteristic gene expression profiles 141. The aforementioned indirect identification strategies provide good supplements and assistance to direct identification strategies (Figure 6).

## Protein degradation methods

Protein degradation based on the phthalimide binding technique 142 and proteolysis targeting chimaeras technique 143 have been reported previously, but these two techniques need to determine the specific degradation ligands of the protein targets, which is not conducive to the target recognition and confirmation of NAPs. Recently, a new protein degradation technology called degradation tag (dTAG) was developed, and it can be used as a tool for drug target identification 131, 144. In this technique, the foreign mutated unnatural protein FKBP12^F36V^ is fused with the protein target to be degraded using clustered regularly interspaced short palindromic repeats (CRISPR), and then the fusion protein is degraded by the dTAG compound, which has good transmembrane properties and selectively binds to FKBP12^F36V^ and E3 ubiquitin ligase 131. dTAG-13 is a highly selective, fast and efficient dTAG molecule, and dTAG technology is a broad-spectrum protein degradation technology that uses a dTAG molecule to accurately degrade broad-spectrum proteins 131. Thus, the candidate protein targets of NAPs are degraded by dTAG molecules for analysis and confirmation. In addition, dTAG can be applied to cells, tissues and organisms, providing a powerful tool for the identification of NAP targets.

## Genomic library screening methods

Genomic library screening is a method for high-throughput drug target identification. This method involves the synthesis of a library of model individuals or cells containing different genes by obtaining individuals or cells with a gene that is knocked out, exhibits reduced expression, is overexpressed or is mutated using molecular biological methods. The expression levels of the protein targets are manipulated by gene knockout to confirm the ligand-binding events. For example, if the cells lacking a specific gene develop resistance to the drug, then the protein encoded by the gene may be the target of the drug. RNA interference (RNAi) and CRISPR-associated protein-9 nuclease (CRISPR-Cas9) are used as gene knockout tools to confirm the interaction between NAPs and targets and to screen drug target genes 145-148. At present, researchers have established at least 13 CRISPR knockout libraries, 3 CRISPRa libraries and 2 CRISPRi libraries that can be used in humans, and these libraries have been used not only for screening NAP targets but also for clinical research 132. Due to the characteristics of genetic susceptibility, stability and simple culture, yeast is a widely used and valuable model organism for studying the mechanisms of diseases and drugs 149. Yeast-based gene deletion and overexpression libraries have been widely used to screen drug targets 150. The combination of these technologies and high-throughput methods has expanded the scope of application of these genomics techniques, enabling hundreds of genes to be silenced in a single experiment, which has become a powerful tool for the indirect identification of NAP targets.

## Differential genomic screening methods and differential proteomic screening methods

The differential genomics strategy obtains its characteristic expression profile by detecting the changes in gene expression levels before and after drug treatment and then examines the related genes and their regulatory networks in the signalling pathway of small molecules to predict the targets of small molecules. Changes in the gene expression profile do not directly determine drug targets, so there are great limitations in applications of these studies to NAP target identification. Recently, a new strategy was proposed to screen small molecule protein targets and explain their modes of action by combining differential genomics and big data analysis. In this strategy, the authors correlated the sensitivity patterns of 481 compounds with ∼19,000 basal transcript levels across 823 different human cancer cell lines and identified selective outlier transcripts 151. By correlating its cytotoxicity with changes in basal transcription levels, as well as performing big data analysis and comparison, a new understanding of the mechanism of action and direct targets of small molecules has been produced. Using this strategy, ML239, which was originally identified in a phenotypic screen for selective cytotoxicity in breast cancer stem-like cells, was shown to most likely act through the activation of fatty acid desaturase 2 (FADS2) 151. Although this strategy has some shortcomings in target identification, as some targets may not show differences at the genetic level or differentially expressed genes do not affect cytotoxicity, it is effective for the unbiased identification of physiological targets of small-molecule compounds, providing a good supplement.

Differential proteomics is used to study the mechanisms of action of NAPs by comparing the relative abundance of proteins in the absence and presence of drugs. The total proteome of cells before and after NAP treatment was extracted for 2D DIGE, and then the information on a large number of proteins exhibiting a change in expression was obtained using quantitative proteomics technology. In recent years, differential proteomics screening methods have been widely used in NAP target identification research 152-157. However, similar to differential genomics, direct protein targets of NAPs are very difficult to identify using differential proteomics. Only a few examples successfully speculate on direct protein targets based on changes in the expression profile 158.

## Cell phenotype-based screening methods

The comparison of cell morphology is a method to quickly screen the targets and modes of action of small molecules based on the morphological changes in organelles in the absence or presence of drug treatment 135. According to the characteristic changes in morphology when exposed to an agent, which are often related to its mechanism of action, chemical-genetic cell morphology profiling was proposed as a promising method 135. Morphobase is an encyclopaedia of cellular morphology consisting of the cell shape changes induced by various compounds in two cancer cell lines 135. Morphobase not only reproduces the "drug-target-phenotype" relationship of drugs with known targets but also predicts unreported mechanisms of action and promotes the discovery of new drug candidates 135. Similarly, a method based on comparing cell morphology combined with differential proteomics screening was developed to predict the targets and modes of action of small-molecule compounds 136. Importantly, this method is very simple and repeatable and only requires the observation of images of stained cells and nuclei in the open field under an ordinary microscope 136. The NCI-60 cell line is a common cancer cell line used to test anticancer drugs, and NCI-60 screening was one of the earliest phenotypic screening systems for cell activity provided by the National Cancer Institute (NCI) in the late 1980s. Using cell phenotype-based screening methods, the natural product halichondrin B was predicted and identified to target the tubulin protein and inhibit microtubule polymerization 159. Combined with a set of established comparison algorithms, NCI-60 screening compares the screening data for any unknown target compound with known drugs to obtain relevant information about its target and mode of action 137. However, as the NCI-60 cell line has been cultured for thousands of generations, its genetic composition and behaviour have changed. New cell models that are closer to the original growth environment are urgently needed. Recently, by integrating image-based phenotypic screening in HeLa cells with high-resolution untargeted metabolomics analysis, a new platform connectivity map (CMAP) was developed that directly predicts the targets and modes of action of bioactive constituents for any complex natural product extract library 160. Using the CMAP platform, quinocinnolinomycins, a new family of NAPs with a unique carbon skeleton that cause endoplasmic reticulum stress, were discovered 160. In conclusion, these high-throughput methods for screening and analysing modes of action of NAPs provide a supplement and reference for identifying protein targets of NAPs.

Organoids are tiny organs that can be cultivated from human stem cells in the laboratory. They have been used to build disease models and may be used to test drugs or even replace damaged tissues in patients in the future 161. In recent years, many studies have found that three-dimensional (3D) organoid culture shows more sensitive characteristics in drug screening, especially antitumour drugs, than 2D culture cell lines 162. Using the advantage of 3D culture in preclinical research may substantially improve our understanding of tumour biology, eliminate poor drug candidates, and be used to screen new cancer-related targets that may be missed in 2D screening 163, 164. Therefore, taking advantage of organoids that simulate the characteristics of the patient's tissues in a 3D culture system *in vitro*, the combination of phenotypic analysis and MS technology to screen protein targets of drugs is helpful for the precise treatment of patients and accurate identification of drug targets. However, the current organoids still lack some structural features related to the function and development of real organs, such as the lack of a vascular system, which is an important structure to obtain energy during the growth and development of human organs. Therefore, organoids cannot be called a “reduced version” of real organs. There are still miniature and simple models lying between animal models and cell models.

## Bioinformatics prediction methods

In addition to comparing the changes in the physical and chemical properties of cell morphology, possible protein targets have been predicted by detecting changes in cell physiology, signalling pathway proteins or gene levels after drug treatment and then using functional analysis and bioinformatics tools. Based on gene expression profile data, a biological application database CMAP was established and updated in 2017 138. According to the principle of graph matching, the CMAP database established the relationships among drugs, genes and diseases through a large database of characteristic gene expression profiles and is widely used in the fields of new use of old drugs, discovery of new drugs, and conjecture of drug mechanism, among others 139. In terms of NAPs, a database platform of NAP gene expression profiles based on the CMAP database was established that could be used for pharmacological activity predictions, target and pathway recognition, and new drug creation based on NAPs 140. This database platform was successfully applied to the natural product nitidine chloride and revealed that it has a new function of blocking α-adrenoceptors to lower blood pressure 140. In addition, using this database platform and the CMAP database, tanshinone IIA was shown to selectively bind the targets protein kinase Cζ (PKCζ) and protein kinase Cε (PKCε), which provided a new insight for understanding the antitumour mechanism of tanshinone IIA 165. Notably, bioinformatics is deeply involved in the entire research process, whether it assists omics technology to screen candidate targets or calculate and predict possible binding sites. With the development of artificial intelligence, this trend will become more evident and even critical.

## Methods for validating protein targets

After screening the candidate protein targets of NAPs, further confirmation is needed. The direct methods use technology consistent with the screening method; for example, the stability of the pure target protein can be tested before and after binding small molecules. If the small molecule and protein are covalently bound, the molecular weight of the protein after binding can also be measured using MS to determine the number of small molecules bound and even analyse the binding sites 131, 166, 167. In addition to these conventional methods, the occurrence of ligand-binding events can be verified by measuring the physical and chemical parameters of small molecules before and after binding to proteins. The commonly used methods are ITC 168, surface plasmon resonance (SPR) 169, spectroscopy 170-173, atomic force microscopy 174, 175, nuclear magnetic resonance (NMR) and other methods 176, 177.

ITC is a method widely used to determine the affinity and thermodynamic parameters of the interaction between soluble proteins and proteins, small-molecule ligands and proteins. In addition, it is also used to determine the number of binding sites between proteins and ligands and the purity of proteins 168, 178. In general, an ITC experiment involves the continuous addition of drugs to the reaction pool containing protein solutions. Each time, a certain number of ligands are added to the protein sample to form a specific number of ligand-protein complexes according to the binding affinity, and the binding affinity is evaluated by monitoring calorie release 179. At present, ITC is the only technique that directly measures the change in enthalpy of intermolecular interactions under isothermal conditions. SPR is a technology developed in the 1990s to detect the interaction between ligands and analytes on biosensor chips based on the surface plasmon resonance principle 180. Surface plasma (SP) refers to an electron dense wave propagating along the metal surface caused by the interaction between free vibrating electrons and photons on the metal surface, which can be excited not only by electrons but also by light waves 181. SPR biosensors have the characteristics of high efficiency, automation, no labelling and high data resolution, which makes them an ideal instrument in the field of drug research, and they can monitor the binding process between NAPs and protein targets in real time. In addition, the kinetic data have been obtained directly using the SPR biosensor, and the affinity constant between NAPs and protein targets can be calculated 169, 182.

Commonly used spectroscopy methods to confirm protein-ligand binding events include fluorescence spectroscopy 170, UV-visible absorption spectroscopy 171, synchronous fluorescence spectroscopy 172 and 3D fluorescence spectroscopy 173. In the fluorescence spectroscopy method, the endogenous fluorescence of proteins is mainly derived from tryptophan, tyrosine and phenylalanine. However, tyrosine and phenylalanine contribute relatively little to the total fluorescence of proteins. Therefore, the fluorescence of tryptophan is usually used to study the interaction between small molecules and proteins 183, 184. When the excitation wavelength is set to 280 nm, the protein shows a strong emission spectrum. Therefore, with the addition of small molecules in the experiment, the fluorescence intensity of proteins decreases gradually, indicating that small molecules quench the fluorescence of proteins 170. In the UV-visible absorption spectroscopy method, because the conformation of the protein determines the microenvironment of the amino acid residues, if the conformation of the protein changes, the microenvironment of the amino acid residues will change, and the UV-visible absorption spectrum will also change 185, 186. UV-visible absorption spectroscopy is a very simple method to explore the structural changes in proteins and is often combined with fluorescence spectroscopy to study the secondary conformational changes after the interaction between target proteins and drugs. Synchronous fluorescence spectrometry is a technique in which the wavelengths of the excitation and emission monochromators are fixed at intervals and scanned at the same time. When the interval between excitation and emission wavelengths is 15 nm, only information about tyrosine (tryptophan) residues is displayed. Therefore, synchronous fluorescence spectroscopy is often used to study the conformational changes in proteins during interactions. The change in microenvironment polarity of the protein chromophore is related to the change in its maximum emission wavelength, so the change in protein conformation induced by ligand binding can be determined by measuring the change in emission wavelength 172. 3D fluorescence spectra reflect the effects of microenvironmental changes on the structure of proteins and provide information about the conformational changes in some proteins; they are also used to study the interactions between proteins and small molecules 173.

The NMR method refers to the physical process in which the energy level of the nucleus with a nonzero spin quantum number undergoes Zeeman splitting under the action of an external magnetic field, resonantly absorbs radio frequency radiation at a specific frequency and transitions from a low-energy state to a high-energy state 176. Based on these principles, NMR uses the information obtained by detecting nuclei in different chemical environments to study the molecular structure, chemical composition, intermolecular interactions and other parameters. Many methods have been developed to study the interactions between proteins and ligands using NMR, and they are divided into two categories: detection of proteins and detection of drug molecules. Early NMR screening of drugs usually used proteins as detection objects and compared the conformational changes in proteins before and after the addition of drug molecules to determine whether small molecules interacted with proteins, which was very suitable for high-throughput drug screening 187. In addition to the detection methods based on known structural proteins, NMR can also be used to study the interactions between proteins with unknown structures and high affinity ligand molecules to obtain the structure of small molecules during ligand interactions 188. Moreover, intracellular NMR spectroscopy can provide *in situ* information about the binding, transport and interaction of drugs and proteins and detect the effectiveness of drugs in a more real environment, which provides great convenience for NAP research and new drug development 189.

## Summary and outlook

**Figure 7:**
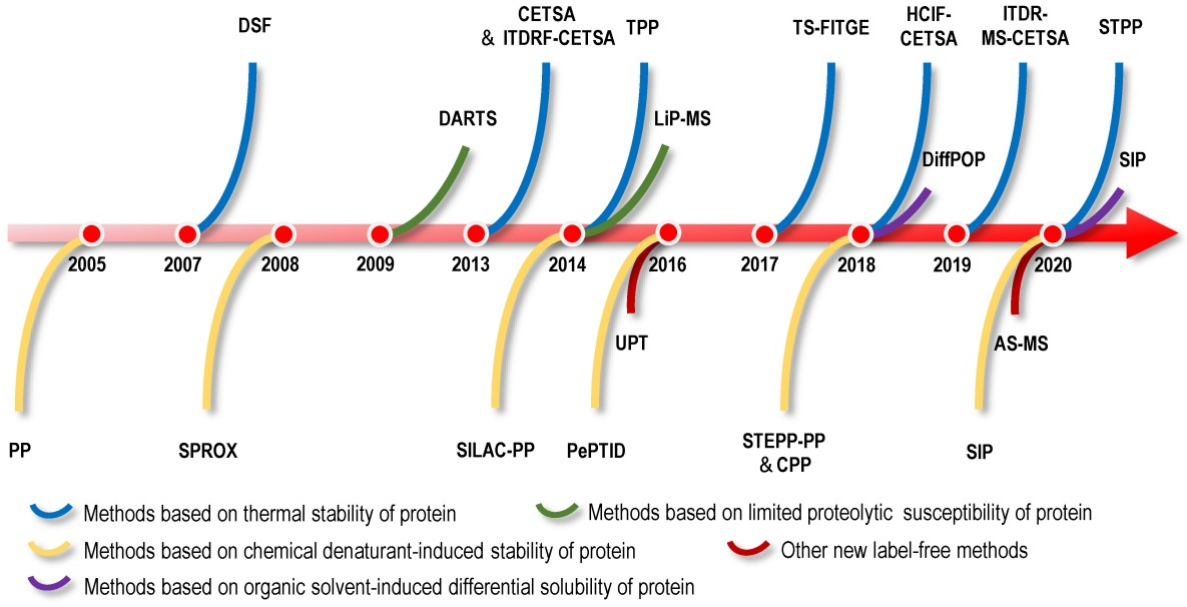
Timeline of the different label-free approaches.

NAPs have been used in practice for many years and exert reliable curative effects. Screening and mining of their protein targets may identify new therapeutic targets. Furthermore, the discovery of new drug targets also provides insights into the structural transformation of NAPs and the design of new drugs. A better method for target identification and confirmation should meet the conditions described below. First, it should maintain the structure and activity of drugs to the greatest extent and not lead to a decrease or loss of activity. Second, it has broad-spectrum applicability, which is suitable not only for high-abundance proteins but also for low-content proteins and for different tissues, cells, and organisms. A variety of factors may affect protein stability, including temperature, pH, salt concentration, organic reagents, surfactants, reductants, and denaturants. After more than ten years of development, protein target identification technologies based on differences in protein stability have gradually matured and are constantly improving (Figure 7). Several unbiased proteomic tools, such as DARTS, SPROX, TPP, and TS-FITGE, were introduced as label-free methods. In particular, TPP and TS-FITGE dissect target engagement in a cellular context via thermally denaturing proteomes in live cells, whereas DARTS and SPROX perturb proteomes after cell lysis. In the past five years, new methods have emerged annually. These new methods are roughly divided into two categories: continuous improvement of existing methods through optimization processes (such as STPP and HCIF-CETSA) and new methods based on different principles (such as SIP and UPT). However, a common limitation of label-free protein target identification methods is the lack of a process for target enrichment; thus, less abundant proteins are difficult to identify. However, this problem can be solved by improving the sensitivity of the instrument and the analysis protocols. In addition, none of the label-free methods cover the entire proteome (Table 1). For example, in target identification based on thermal stability, the change in the thermal stability of the protein may not be detected even if the protein target specifically binds to the drug molecule. This phenomenon often occurs in the presence of the endogenous ligand of the protein, which forms a large protein complex with the protein or is embedded in the membrane.

At present, the method of direct affinity enrichment based on chemical proteomics is still the mainstream scheme for the identification of NAP targets, while the development of biophysical technology provides a new choice for the identification of unlabelled and unbiased targets. In addition, the phenotype-based indirect target identification strategy provides a good supplement and assistance to overcome the shortcomings of the direct strategy. Notably, the protein target information obtained using both direct and indirect strategies is potential or speculative, and sometimes hundreds of potential protein targets may be identified, which must be further verified at the level of physical binding and physiological function. Therefore, a variety of strategies can be simultaneously used for target identification to reduce the workload. With the development of and advances in proteomics, genomics, bioinformatics and MS, additional new technologies will integrate the advantages of different technologies and reduce the cost and time of detection. These new technologies will not only be applied to recognize NAP targets but also clarify the mechanisms of action and toxicity of NAPs, which will provide an important method for solving the problems of new drug research and development, drug mechanism research, human disease marker recognition and other issues.

## Figures and Tables

**Table 1:** Pros and cons of different label‐free target identification approaches

Category and working principle	Methods	Sample to perturb	Readout coupled for identification	Advantages	Limitations	Proteome coverage
Based on the differences in the thermal stability of protein targets	DSF	Purified protein	qPCR	1. Easy to implement, flexible setup and versatile formats; 2. Rapid drug targets screening.	1. Need purified proteins and low throughput of target engagement; 2. Many disturbance factors to affect the results; 3. The binding of the ligand may not protect the protein against thermal denaturation.	-
	CETSA	Living cells and tissues	WB	1. Easy to implement, flexible setup and versatile formats; 2. Versatile sample types including living cells and tissues; 3. Coupled to many different readouts; 4. Advantageous to compounds that need cellular metabolism for activation; 5. Could reveal downstream effect when dosed in live cells.	1. Low throughput of target engagement; 2. Not suitable for low expression of proteins; 3. Difficult to detect some proteins containing unfolded biding sites; 4. The availability of antibodies.	-
	TPP (MS-CETSA)	Living cells and tissues	MS (TMT)	1. Versatile sample types including living cells and tissues; 2. Coupled to many different readouts; 3. Advantageous to compounds that need cellular metabolism for activation; 4. Could reveal downstream effect when dosed in live cells; 5. high throughput of target engagement.	1. Inaccurate for some proteins have very low or high *T*m; 2. Difficult to detect some proteins containing unfolded biding sites; 3. Fractionation before LC-MS/MS analysis is required; 4. High cost and labour; 5. Difficult for membrane proteins because their stability and solubility.	~5000-8000 proteins for proteome samples
	ITDR-MS-CETSA	Living cells and tissues	MS (TMT)	same as above 1-5; 6. Could report the extent of target occupancy; 7. Only one temperature with significant precipitation is analysed.	Same as above 3-4.	~5000-8000 proteins for proteome samples
	STPP	Living cells and tissues	MS (Isotopical dimethyl)	1. Rapid labelling, accurate quantification and cost-effective; 2. Only 2-3 temperatures with significant precipitation are analysed; 3. One dimensional LC-MS/MS; 4. Versatile sample types including living cells and tissues.	Suitable for the initial screening of the protein targets of ligands and need further verification.	~2000 proteins for proteome samples
	TS-FITGE (2DE-CETSA)	Living cells and tissues	2D fluorescence gel +MS (Cy3/Cy5)	1. Economical, fluorescent dyes to proteins; 2. Proteoform differentiation is straightforward; 3. Versatile sample types including living cells and tissues.	1. Complex samples to measure and analyse; 2. Proteome coverage is limited by finite region of the gel.	~1000 proteins for proteome samples
	HCIF-CETSA	Living cells and tissues	Fluorescence imaging	Allow for distinguishing target engagement in specific cell types and heterogeneous samples *in situ*.	1. Low throughput of target engagement; 2. The availability of antibodies; 3. The epitope after the protein is partially denatured during the fixation process.	-
Based on the difference in chemical denaturant-induced stability of protein targets	PP	Lysates	WB	1. Easy to implement, flexible setup; 2. Possible to obtain thermodynamic parameters.	1. Only applicable to cell lysates; 2. Not suitable for low-abundance proteins; 3. Complex chemical denaturant titration to preparation; 4. Low throughput of target engagement; 5. The availability of antibodies.	-
	SILAC-PP	Lysates	SDS-PAGE + MS (SILAC)	Same as above 1-2; 3. The use of bottom-up proteomics improve the assay sensitivity.	Same as above 1-4; 5. More suitable as a validation rather than discovery strategy. 6. Proteome coverage is limited by finite region of the gel.	~1000 proteins for proteome samples
	CPP	Lysates	MS (TMT)	same as above 1-2 3. Possible to obtain thermodynamic parameters; 4. Not rely on detecting specific amino acid-containing peptides.	Same as above 1-4; 5. High false positive rates; 6. Complex chemical denaturant titration to preparation.	~1000 proteins for proteome samples
	SPROX	Lysates	MS (iTRAQ; SILAC; TMT)	1. Peptide-level resolution and domain-level binding information; 2. Possible to obtain thermodynamic parameters; 3. Irreversible reaction of protein oxidation; 4. Can provide flexibility for down-stream quantitative proteomics.	1. Only applicable to the proteins containing methionine residue; 2. Complex chemical denaturant titration to preparation; 3. Need a relatively large amount of starting material.	~1000 proteins for proteome samples
Based on the differential limited proteolytic susceptibility of protein targets	DARTS	Lysate	SDS-PAGE + MS	1. Could analyse true interactions with low affinity; 2. Cell lysates can be applicable for target identification.	1. Limited to relatively higher abundance proteins; 2. Proteolysis is a multifactorial event, influenced by choice of protease, lysis buffer, and detergent conditions; 3. Should be validated with steps for preparation and proteolysis of cell lysates.	~5000-6000 proteins for proteome samples
	LiP-MS	Lysate	MS (STEPP)	Peptide-level resolution and domain-level binding information.	1. Complex samples to measure and analyse; 2. limited to relatively higher abundance proteins; 3. Binding affinity and target proteins susceptibility to proteolysis are limiting factors; 4. Not suitable for proteins that are very sensitive or resistant to proteolysis.	~5000-6000 proteins for proteome samples
Based on the organic solvent-induced difference in the solubility of protein targets	SIP	Lysate	MS (Isotopical dimethyl)	1. Relatively easy to implement; 2. Determine the drug-protein affinity in total cell lysate using dose-response assay.	1. Relatively low throughput of target engagement; 2. The setting of judgment conditions in data analysis is subjective; 3. Hard to detect proteins with higher solubility in organic solvents.	~1000-2000 proteins for proteome samples
Based on weak molecular interactions of bait-molecule and polymeric surface	UPT	Lysate	SDS-PAGE + MS	1. Underivatized bait-molecule and target Identification trough affinity-based target enrichment; 2. Rapid target profiling of multiple compounds, saving time and cost; 3. Low false positive rate.	Certain highly water soluble and/or highly hydrophobic compounds may not get immobilized on current available matrix.	-
Based on size exclusion chromatography	AS-MS	Purified protein	LC/MS	1. Label- and immobilization-free; 2. Compounds with a low dissociation constant can be identified; 3. Could identify soluble and membrane proteins; 4. High throughput of target engagement.	Need to fix the molecule, experimental procedures are relatively cumbersome.	-

## Abbreviations

2D: two-dimensional
2D DIGE: two-dimensional gel electrophoresis
2DE-CETSA (or TS-FITGE): thermal stability shift-based fluorescence difference in two-dimensional gel electrophoresis
3D: three-dimensional
ABPP: activity-based protein profiling
ABPs: activity-based probes
AfBPP: affinity-based protein profiling
AS-MS: affinity selection-mass spectrometry
CCCP: compound-centric chemical proteomics
CETSA: cellular context thermal shift assay
Cm: midpoint denaturant concentration
CMAP: connectivity map
CPP: chemical denaturant and protein precipitation
CREB: cAMP-response element binding protein
CRISPR: clustered regularly interspaced short palindromic repeats
CRL4: cullin ring-finger ubiquitin ligase 4
Cy3: cyanine-3
Cy5: cyanine-5
DARTS: drug affinity responsive target stability
DiffPOP: differential precipitation of proteins
DSF: differential scanning fluorometry
E2: ubiquitin-conjugating enzymes E2
FASP: the filter-aided sample preparation
FITGE: fluorescence difference in two-dimensional gel electrophoresis
FKBP: FK506-binding protein
gRNA: guide RNA
HCIF-CETSA: immunofluorescence-cellular context thermal shift assay
ITDRF-CETSA: isothermal dose-response fingerprint-cellular context thermal shift assay
ITC: isothermal titration calorimetry
ITDR-MS-CETSA: isothermal dose‐response mass spectrometry-cellular context thermal shift assay
iTRAQ: isobaric tags for relative and absolute quantitation
K: lysine residue
LysC: lysyl endopeptidase
LC/MS: liquid chromatography-mass spectrometry
LC-MS/MS: liquid chromatography-tandem mass spectrometry
LiP: limited proteolysis
LiP-MS: limited proteolysis-mass spectrometry
MS: mass spectrometry
MS-CETSA (or TPP): thermal proteome profiling-cellular context thermal shift assay
NAP: natural active product
NMR: nuclear magnetic resonance
ORF: open reading frame
PCCs: Pearson's correlation coefficient
PePTID: pulse proteolysis and precipitation for target identification
PP: pulse proteolysis
qPCR: quantitative real-time PCR
R: arginine residue
RNAi: RNA interference
SDS-PAGE: sodium dodecyl sulfate-polyacrylamide gel electrophoresis
SILAC: stable isotope labelling with amino acids in cell culture
SILAC-PP: stable isotope labelling with amino acids in cell culture-pulse proteolysis
SILAC-SPROX: stable isotope labelling with amino acids in cell culture-stability of proteins from rates of oxidation
SIP: solvent-induced protein precipitation
SPR: surface plasmon resonance
SPROX: stability of proteins from rates of oxidation
STEPP: semitryptic peptide enrichment strategy for proteolysis procedures
STEPP-PP: semitryptic peptide enrichment strategy for proteolysis procedures-pulse proteolysis
STPP: simplified thermal proteome profiling approach
TCA: trichloroacetic acid
Tm: melting temperature
TMT: tandem mass tag
Ub: ubiquitin
UPT: unique polymer technology
UV: ultraviolet
WB: western blotting
